# Application of glyphosate or paraquat before the emergence of maize plants (*Zea mays* L.) had no detectable effect on the rhizosphere or endophyte bacterial community structure under the tested field conditions

**DOI:** 10.3389/fmicb.2026.1892064

**Published:** 2026-08-05

**Authors:** Claudia E. Aceves-Suriano, Ana Lilia Toriz-Nava, Simon Fonteyne, Nele Verhulst, Bram Govaerts, Gabriela Medina-Pérez, Marco L. Luna-Guido, Yendi E. Navarro-Noya, Jesús Bernardino Velázquez-Fernández, Luc Dendooven

**Affiliations:** 1 Laboratory of Soil Ecology, Cinvestav, Mexico City, Mexico; 2 Centro de Investigación en Ciencias Biológicas, Universidad Autónoma de Tlaxcala, Tlaxcala, Mexico; 3 International Maize and Wheat Improvement Center (CIMMYT), El Batán, Texcoco, Mexico; 4 School of Integrative Plant Science, Cornell University, Ithaca, NY, United States; 5 Instituto de Ciencias Agropecuarias, Universidad Autónoma del Estado de Hidalgo, Pachuca, Mexico

**Keywords:** bulk soil, maize plant compartments, microbiome, nonselective herbicide, putative metabolic functions, rhizosphere

## Abstract

Herbicides are applied in agricultural practices to manage weeds. How these herbicides applied before maize emergence (*Zea mays* L.) might affect the rhizosphere and endophytic bacterial community is still largely unknown. In this study, plots were treated with 400 g paraquat ha^−1^ (Gramoxone^®^, paraquat ion 20% w/w), 1,089 g glyphosate ha^−1^ (Faena^®^, potassium salt of glyphosate 446 g/L) or weeds were hand removed before maize emerged and the bacterial community was determined in the bulk soil at the start of the experiment, in the bulk soil, rhizosphere, roots and stem at flowering (after 101 days), in the rhizosphere, roots, stem and grains at flowering (after 166 days) and in harvested grains dried for 24 days. Application of paraquat or glyphosate had no significant effect on the bacterial community structures and its putative metabolic functions compared to those in the manual weed-removed control plots. *Haliangium_463188* was enriched in soil when glyphosate was applied and *Flavobacterium* in plots treated with paraquat. The bacterial community and its metabolic functions was different between the bulk soil, rhizosphere, root and stem, while it was similar in the stem and grains. Two phylotypes, i.e., an unidentified Alphaproteobacteria and uncultured member of the *Marinilabiliaceae* (*JC017*), dominated in the stem and grains of the maize plants (relative abundance > 95%). Many putative biosynthesis pathways were strongly enriched in the maize stem compared to the roots. It was found that application of glyphosate or paraquat before maize emergence had no significant effect on the soil, rhizosphere, root, stem or grain bacterial community structure. However, there was a clear change in the bacterial community from the rhizosphere to the roots, stem and grains of the maize plants and an unidentified Alphaproteobacteria and uncultured member of the *Marinilabiliaceae*, i.e., *JC017*, became highly dominant.

## 1 Introduction

Endophytes are microorganisms that live inside plants without harming their host (Wu et al., 2021). They play an important role in plant development by producing hormones, antagonizing pathogens, alleviating fungal and bacterial stress, and increasing resistance to abiotic stress (Lata et al., 2018; Muthu Narayanan et al., 2022; Anand et al., 2023). As such, endophytes are important in crop production (Watts et al., 2023). Different biotic and abiotic factors, as well as host genetic factors, shape the diversity, community structure and relative abundance of endophytes (Schönrogge et al., 2022). Agricultural practices affect endophytes of different plants in different ways. For instance, Castillo-González et al. (2024) studied the effect of conventionally managed vs. organically managed coffee plants (*Coffea arabica* L.) on the fungal leaf endophytes and concluded that conventionally managed plants had a higher taxonomic endophytes diversity, while the relative abundance of putative mycoparasites was larger in the organically managed ones. Arevalo Alvarenga et al. (2024) found that mowing height and N fertilization rate had a significant effect on the diversity of endophytic diazotrophs in CitraBlue St. Augustinegrass [*Stenotaphrum secundatum* (Walter) Kuntze]. In a previous study, we found that nitrogen fertilizer application changed the root endophyte bacterial microbiome in maize plants, but not in the stem or rhizosphere soil (Miranda-Carrazco et al., 2022).

Weeds must be managed in intensive agriculture as they compete with crops for water, nutrients and sunlight (Kaur et al., 2018). Herbicides are chemical products used worldwide to control weeds and are vital in modern agriculture (Parven et al., 2025). Glyphosate and paraquat are two of the most applied herbicides worldwide.

Glyphosate, the most used herbicide in the world (Benbrook, 2016), inhibits the shikimate pathway, which is involved in essential amino acids production tyrosine, tryptophan and phenylalanine (Hertel et al., 2021). This pathway is found in bacteria, fungi, algae and plants, but not yet in animals (Shende et al., 2024). Glyphosate might affect non-target animals, such as bees visiting flowering plants through plant pollen (Zioga et al., 2022). Paraquat is one of the most used herbicides in the world (Nazish et al., 2022), but also considered a risk to the environment and human health (US-EPA–United States Environmental Protection Agency, 2025). Paraquat is a widely used herbicide due to its rapid action and being active only by spraying directly onto plants (Bromilow, 2004). It generates free radicals that cause damage to the cell membranes (Charif et al., 2026). Paraquat generates oxidative stress in plants exceeding the capacity of the plant to dissipate the amount of O_2_ and H_2_O_2_ generated so that the cell dies (Nazish et al., 2022; Charif et al., 2026). Paraquat can also affect human health and has been related to lung injury (Gao et al., 2020; Liu et al., 2022), pulmonary fibrosis (Liu et al., 2022; Sun et al., 2020) and Parkinson’s disease as it induces oxidative stress in the human brain (See et al., 2022).

The mobility of herbicides and their possible effect on the microbial community will be defined by soil and herbicide characteristics (e.g., Sibalekile et al., 2025). Both glyphosate and paraquat are highly soluble in water but they are characterized by a low mobility and strong adsorption. For instance, the Freundlich distribution coefficient (*Kf*) reflecting the adsorption capacity, is high for both glyphosate (ranging from 6.1 to 259.5 L/kg Skeff et al., 2018) and paraquat (ranging from 18 L/kg in sandy and 787 L/kg in clay soils) (Amondham et al., 2006). As such, both will be strongly adsorbed in soil. Glyphosate is normally biodegraded rapidly in soil with an estimated half-live time ranging from 7 days (Li et al., 2025) to 60 days (Aluffi et al., 2025) depending on environmental conditions (Li et al., 2025). Microorganisms degrade it by cleaving the C-N and C-P bounds, and using the products as P, N and C sources (Chen et al., 2022). The half-life time of paraquat, however, can be much larger and can be up to > 6 years (Alexander, 1999) and no clear pathway for its degradation has been described yet. When recommended doses of herbicides were applied, no changes in the microbial community were detected in soil (Dennis et al., 2018) and roots of maize and soybean (Kepler et al., 2020). However, the effect of both herbicides on specific microbial groups will be highly variable as some bacteria and fungi can degrade glyphosate while others are inhibited by it (Sibalekile et al., 2025).

Herbicides are used to target unwanted weeds, and non-selective herbicides, such as glyphosate and paraquat, are applied before the emergence of the cultivated crops (Parven et al., 2025). How herbicides applied before cultivated plants emerge might affect the rhizosphere bacterial community and endophytes of crops, such as maize (*Zea mays* L.), remains largely unknown. Ueno et al. (2024) stated that herbicides might affect plant symbionts and as such the interaction between them and the host thereby altering the way the host deals with environmental stresses. On the one hand, some studies have shown that glyphosate application can alter the composition and diversity of soil bacterial communities, affecting microbial groups involved in nutrient cycling and plant health (Chávez-Ortiz et al., 2022). Similarly, paraquat has been found to affect negatively the microbial metabolic activities and reduce the beneficial microorganisms in soil (Teke et al., 2024). Herbicides, such as glyphosate and paraquat, might not only affect soil microorganisms but also plant endophytes. For instance, Mohamed et al. (2021) inoculated Arabian pea or pitch trefoil plants (*Bituminaria bituminosa* (L.) C.H.Stirt.) with four N_2_-fixing symbiotic bacteria, i.e., *Pantoea agglomerans, Rhizobium nepotum, R. radiobacter* and *R. tibeticum* and applied different concentrations of glyphosate and paraquat. They found that both herbicides decreased the number, size weight of the nodules, N_2_ content of *Bituminaria bituminosa* and symbiotic efficiency of the four different nitrogen-fixing bacteria studied after 6 months, and the effect increased with increased concentration applied. On the other hand, some studies reported that recommended doses of herbicides, such as glyphosate and paraquat, did not alter the soil bacterial, archaeal or nematode community composition under controlled conditions (Dennis et al., 2018). As such, a possible effect of the use of the herbicides paraquat and glyphosate on the bacterial endophytes of maize (*Zea mays* L.), an important crop worldwide, should be investigated. Therefore, we investigated how the use of glyphosate and paraquat to control weeds in a field experiment affected the bacterial communities in the bulk soil, rhizosphere and endosphere of non-targeted maize.

The effect of the herbicide’s glyphosate and paraquat on maize development was investigated in a field experiment at CIMMYT’s experimental site in the central highlands of Mexico. We sampled the soil before application of the herbicides, the bulk and rhizosphere soil, and maize roots at flowering, and the rhizosphere, roots, stem and grains at harvest. The bacterial community was characterized by amplification and sequencing of the 16S rRNA gene. The objectives of this study were (i) how did the application of the herbicides glyphosate or paraquat affect the bacterial community and their putative metabolic functions in the soil, and rhizosphere, roots, stem and grains of maize plants compared to an manual weed-removed control, (ii) how did the bacterial communities and their putative metabolic functions change from the soil to the rhizosphere, roots, stem and finally to the grains, and (iii) did the bacterial communities and their putative metabolic functions change in the soil, rhizosphere and roots during plant development, i.e., at different growth stages (sowing, flowering and harvest). It was hypothesized that the application of the herbicides before planting would have a limited effect on the bacterial communities and their putative metabolic functions, but that the bacterial community and their putative metabolic functions in soil, rhizosphere and maize plants would clearly be different independent of the application of the herbicides. This research aimed to study the effect of application of the glyphosate or paraquat on the bacterial communities associated with maize, i.e., bulk soil, rhizosphere and endophytic compartments, when emerging weeds had been treated previously with these herbicides. Investigating a possible effect of the herbicides glyphosate and paraquat on bacterial communities is essential for understanding how these chemicals might indirectly affect endophyte bacterial communities within crops and, ultimately, the health and productivity of agricultural systems.

## 2 Materials and methods

### 2.1 Experimental site, soil, and maize plant collection

The research station El Batán is located in Texcoco (2,240 masl; 19.318^o^ N, 98.508^o^ W) in the semiarid, subtropical highlands of central Mexico. The monthly average temperatures ranged between 11.7 and 18.2 °C with yearly average of 15.5 °C, while monthly average rainfall ranges between 7 and 120 mm with yearly average of 651 mm (1991–2018).

Soil and plants used in this study were collected from a field experiment that investigated the effect of herbicide use on production of maize. The experiment was sampled in the second year of the field trial. The year before the experiment was started, glyphosate was applied before planting of the crop. In the first year of the field trial with maize, the same herbicides concentrations were used, and the same treatments were applied to the same plots as in the second year, i.e., the year samples were taken for this study. The experimental design and sampling protocol is given in Supplementary Figure S1. The experiment had a randomized complete block design (3 × 5 m) with three replicates (*n* = 3) and six treatments that investigated the effect of different herbicides on crop yields. Only three treatments, i.e., weeds treated with glyphosate or paraquat, or removed manually, were included in this study (*n* = 3). Soil sampling was done in the second year of the experiment, and herbicide treatments were the same as in the previous season. A first soil sample was taken on the 5th of June 2022 just before planting, and application of the herbicides and fertilizer. Ten soil cores (∅ 2 cm, 0–20 cm depth) were taken from the central two rows of each plot and pooled. As such, nine soil samples were obtained and characterized.

Just after collecting the soil samples, maize was sown (6th of June 2022), and the herbicides were applied 2 days later (8th of June 2022). The maize variety used in this experiment was a non-genetically modified hybrid. The herbicides were applied with a backpack sprayer as is the custom locally. In a first application, plots were treated with 1,089 g glyphosate ha^−1^ (Faena^®^, potassium salt of glyphosate 446 g/L), while in a second with 400 g paraquat ha^−1^ (Gramoxone^®^, paraquat ion 20% w/w) and in a third treatment which served as control no herbicide was applied, but the weeds were removed twice manually using a hand hoe. The herbicides were applied at the rates as stipulated by the manufacturers and applied directly to the weeds. Plants were fertilized with urea at 100 kg N ha^−1^ and P_2_O_5_ at 30 kg P ha^−1^ as in normal agricultural practices in the region. The maize plants emerged on the 10th of June 2022.

On the 14th of September 2022, a week after flowering, three complete maize plants were sampled from each plot (*n* = 3) and treatment (*n* = 3) together with their associated rhizosphere. The collected maize plants were shaken by hand for 10 min. The soil that did not remain fixed to the roots was discarded. The remaining soil fixed to the roots, i.e., the rhizosphere soil, was collected by hand-shaking the roots in 1 L sterile 0.9% NaCl solution for 10 min (Navarro-Noya et al., 2022). The soil suspension was centrifuged at 150 *g* at room temperature for 15 min. Bulk soil was sampled from between the maize plantlet rows. Bulk soil, the rhizosphere and the maize roots were extracted for DNA as described below. Before harvest, on the 18th of November, four maize plants were sampled from each plot (*n* = 3) and treatment (*n* = 3) together with their rhizosphere. The rhizosphere and the maize roots, stem and grains were extracted for DNA as described below. The maize roots were so extensive that no bulk soil could be sampled, i.e., little or no soil without roots remained. Additionally, maize grains were dried for 24 days and extracted for DNA as described below.

Plant tissues (roots, grains and stems) were immersed sequentially in hydrogen peroxide (H_2_O_2_) for 10 min, in 1 M sodium hypochlorite (NaOCl) for 15 min, in 70% ethanol for 10 min followed by two rinses with sterile distilled water (Davoudpour et al., 2020). The DNA was extracted from the water of the final rinse to confirm a successful surface sterilization. No DNA was detected in the water indicating the effective removal of external microbial contaminants in each plant tissue.

### 2.2 Soil characterization

The sand content of the clay loam soil was 400 g kg^−1^, the clay content 310 g kg^−1^ and the loam content 290 g kg^−1^. The soil had pH 6.5, electrolytic conductivity (EC) 0.52 dS m^−1^, a total C content of 10.3 g kg^−1^ and total N content of 0.84 g kg^−1^. The pH was determined by mixing a soil sample with distilled water in a 1:2.5 ratio and pH was measured with a potentiometer Mettler Toledo® Model S220 (New York, USA) Thomas (1996). The EC was measured in the supernatant with a conductometer Mettler Toledo^®^ Model S220 (New York, USA) (Rhoades et al., 1989). The soil particle size distribution was determined by the hydrometer method (Gee and Bauder, 1986). Total C was determined with a Thermo Scientific™ FlashSmart™ Elemental Analyzer (Waltham, Massachusetts, USA) following the standard protocol given by the manufacturer. Total N was measured by the Kjeldahl method using concentrated H_2_SO_4_, K_2_SO_4_ and HgO to digest the sample (Bremner, 1996). Each characterization was done separately on a sub-sample from each treatment applied in the field (*n* = 3) and each plot (*n* = 3).

### 2.3 Soil, rhizosphere and maize plant DNA extraction

The soil, rhizosphere, roots, stem and grains samples were collected and extracted for DNA. A 300-mg sub-sample of soil and rhizosphere were extracted for DNA using the Qiagen DNeasy PowerSoil Pro Kit following the manual instructions (Qiagen, Venlo, The Netherlands). The roots, stem and grains samples were extracted for DNA as follows. First, the tissue was disinfected and macerated with liquid nitrogen. The DNA was extracted using the cetyltrimethylammonium bromide (CTAB) modified extraction method. Plant and grain tissue was lysed with a CTAB solution and incubated at 65 °C in a Thermoblock Thermo Scientific dry bath heating (Thermo Fisher Scientific, Waltham, Massachusetts, United States) shaken every 3 min for 30 min. 3 μL mercaptoethanol was added to the solution, incubated at 65 °C, shaken every 10 min for 1 h, and centrifuged at 10,000 RPM and 4 °C for 8 min. The supernatant was added to a new tube, 400 μL C_2_H_7_NO_2_ 7.5 M was added, and the solution centrifuged at 10,000 RPM for 8 min. The supernatant was transferred to a new tube, 700 μL chloroform: octanol 24:1 was added, and the solution was shaking until homogenized. The homogenized solution was centrifuged at 10,000 RPM and 4 °C for 8 min. The centrifugation was repeated until the sample was transparent. The supernatant was transferred to a new tube containing 600 μL cold absolute ethanol and left to stand at −20 °C overnight. The solution was centrifuged at 10,000 RPM and 4 °C for 8 min and the supernatant discarded. The obtained pellet was cleaned with 300 μL cold ethanol 70% and centrifuged at 10,000 RPM and 4 °C for 8 min. The supernatant was discarded, the pellet dried for 25 min and 50 μL deionized sterile water added. PCR negative and positive controls were used to ensure the quality of the amplified DNA. All the solutions used in the experiment were sterilized before use and all extraction procedures were done under sterile conditions in a fume cupboard. No DNA could be extracted from the solutions used.

### 2.4 PCR amplification of 16S rRNA

The extracted soil, rhizosphere, root, stem and grain DNA was used as template for a polymerase chain reaction (PCR) amplification. The high-throughput sequencing primers used in this study were the universal bacteria primers 341F (5′- CCTACGGGIGGCWGCAG −3′) and 805R (5’-GACTACHVGGGTATCTAATCC -3′) that target the V3-V4 bacterial regions (Herlemann et al., 2011). In this step, PCR negative and positive controls were used to ensure the DNA amplified quality. Amplicons of 16S rRNA were purified with the DNA clean kit (Zymo Research, Irvine, CA, United States). Excess primers were cut and purified with FastGene Gel/PCR Extraction Kit (Nippon Genetics, Co., Ltd., Japan) after the DNA being recuperated from agarose gel. The DNA was quantified with the PicoGreen dsDNA Assay Kit (Invitrogen, Carlsbad, United States) using a NanoDrop 3300 Fluorospectrometer (Thermo Fisher Scientific, Waltham, MA, United States). The DNA was sequenced by Macrogen Inc. (DNA Sequencing Service, Seoul, Korea) using a MiSeq 2 × 300 PE (Illumina, San Diego, California, United States) performed.

### 2.5 Bioinformatic analysis

The raw fastq files obtained from the DNA sequencing were processed with “Quantitative Insights Into Microbial Ecology” v 2023.9 (QIIME 2) (Bolyen et al., 2019). The barcode extraction and demultiplexing were completed with the *cutadapt* plugin of QIIME 2 (Martin, 2011). The primer removal, merging of reads, chimera removal, inference of amplicon sequence variants (ASVs) and dereplication were performed with DADA2 in a QIIME2 environment (Callahan et al., 2016). Low quality sequences were removed (< 20 Phred score). The taxonomy was assigned to ASVs using q2-feature-classifier plugin (Bokulich et al., 2018) classify-sklearn against Greengenes2 (McDonald et al., 2024) trained database for V3-V4 region, into QIIME2 environment.

The feature tables and representative sequences generated by DADA2 were used as inputs for metabolic functions prediction. The functional profile of the bacterial community was putative with the “Phylogenetic Investigation of Communities by Reconstruction of Unobserved States” (PICRUSt2) (Douglas et al., 2020) (https://github.com/picrust/picrust2, accessed in November 2024). The pathways based on KEGG Orthology (KO) numbers were annotated with the “Kyoto Encyclopedia of Genes and Genomes” (KEGG). The putative functional profile was also determined with Functional Annotation of Prokaryotic Taxa FAPROTAX v 1.2.10, a database that maps prokaryotic clades with relevant ecological functions. An implicit assumption of FAPROTAX is that if all cultured members of a clade can perform a particular function (at the time of the publication cited), then all will. As more organisms are being cultured, some of these generalizations may turn out to be incorrect.

### 2.6 Statistical analysis

All statistical analyses were done in R version 4.4.1 (2024-06-14) (The R Foundation for Statistical Computing, 2024) within the RStudio environment (Version 2023.09.0 + 463). Alpha diversity of the bacterial community was determined based on the Hill numbers at different *q* orders (*q* = 0, 1 and 2) (Chao et al., 2010). The Hill number at *q* = 0 gives the species richness, *q* = 1 is the Shannon entropy and denotes frequently occurring species and *q* = 2 is the inverse Simpson and characterizes dominant species (Chao et al., 2014) and they were calculated with the HillR package v. 0.5.3 (Li, 2023). A non-parametric analysis (t1way test of the WRS2 package, v. 1.1–6, Mair et al., 2024) was used to determine the effect of the herbicide application, i.e., manual weed-removed control plots, and glyphosate and paraquat treated plots, and the compartments, i.e., bulk soil, rhizosphere, roots, stem and grains, on the Hill numbers.

Ordination (principal component analysis, PCA) and multivariate comparison (perMANOVA) were done with converted sequence data using the centered log-ratio transformation test returned by the aldex.clr argument ALDEx2 (v. 1.36.0) (Gloor et al., 2023). A block structure with plot was used in the perMANOVA analysis [setBlocks(perm) < − with(data, plot)] with 999 permutations [perm<− how(nperm = 999)]. The pairwiseAdonis’ version 0.4.1 was used for multilevel pairwise comparison using adonis() from package ‘vegan’. The function returns adjusted *p*-values using p.adjust(). The FactoMineR (v. 2.11) package (Husson et al., 2024) was used for the PCA and the vegan (v. 2.5–6) package to determine the homogeneity of groups dispersions (Oksanen et al., 2024).

The effect size which is defined as the difference between groups divided by the maximum dispersion within group A or B was calculated after a centered log-ratio transformation with the aldex.ttest function of the ALDEx2 package (v. 1.36.0) (Gloor et al., 2023). The effect size was calculated comparing the effect of herbicide application, i.e., weeds removed manually or plots applied with glyphosate or paraquat, on the bacterial groups in the bulk soil, rhizosphere, roots, stem and grains at flowering and/or harvest. The effect size was also calculated comparing the bacterial groups in the bulk soil, rhizosphere, roots, stem, and grains, when the weeds were removed manually, or plots were applied with glyphosate or paraquat at flowering and/or harvest. Additionally, the effect of time and plots on the relative abundance of the bacterial groups and putative metabolic pathways was also computed.

### 2.7 Sequence database deposition

The raw sequences dataset was deposited in the NCBI-SRA (Sequence Read Archive) Temporary Submission ID SUB14875009 with accession number PRJNA1191893 and Release date: 2024-11-30 (https://www.ncbi.nlm.nih.gov/sra/PRJNA1191893).

## 3 Results

### 3.1 Bacterial Hill numbers

The Hill numbers of the bacterial groups up to the taxonomic level of genus at *q* = 0 (richness) ranged between 202 and 249 in the bulk soil, 316 and 371 in the rhizosphere, 100 and 138 in the roots, 2 and 34 in the stem, 12 and 18 in the grains sampled at harvest, and 5 and 17 in the grains dried for 24 days (Supplementary Figure S2). Similar differences were detected for the number of frequent (Hill number *q* = 1) and dominant bacterial genera (Hill number *q* = 2).

The Hill numbers at *q* = 0, 1 and 2 were not affected significantly by herbicide applications in the bulk soil, rhizosphere, and roots, stem and grains of the maize plants (Supplementary Table S1). However, the Hill numbers at *q* = 0 were significantly different in the bulk soil, rhizosphere, roots, stem and grains, with the highest values found in the rhizosphere and the lowest in the grains (*p* < 0.05). The Hill number at *q* = 1 was significantly higher in the roots than in the stem, but lower than in the rhizosphere and bulk soil, while the Hill number at *q* = 2 was significantly higher in the soil and rhizosphere than in the roots (*p* < 0.05) (Supplementary Figure S2). Time of sampling had no significant effect on the Hill numbers (Data not shown).

The effect of application of glyphosate on the beta diversity of the bacterial groups compared to the manual weed-removed control plots was very similar to that of the application of paraquat (No data shown). The effect of maize cultivation on the beta diversity of the bacterial groups was very similar in the maize plants cultivated in the manual weed-removed control plots and those applied with glyphosate or paraquat (No data shown). It was mostly explained by turnover of bacterial groups (68% mean of all data) although nestedness, i.e., species gain or loss without substitution, was also detected (32%).

### 3.2 The bacterial community

**Figure 1:**
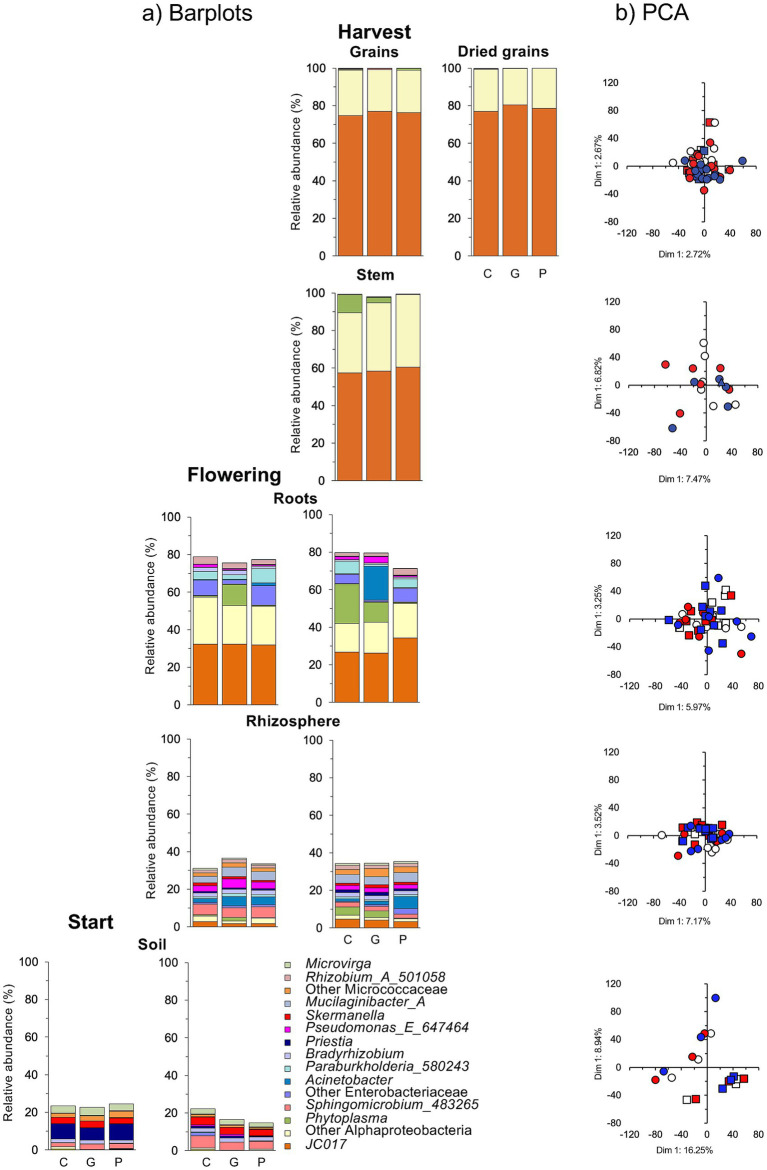
**(a)** Barplots with the relative abundance (%) of the 15 most abundant bacterial groups assigned up to the taxonomic level of genus in the soil, and rhizosphere, roots, stem and grains of maize plants (*Zea mays* L.) when weeds were removed manually (C) or plots were treated with glyphosate (G) or paraquat (P). **(b)** Principal component analysis (PCA) with all bacterial groups assigned up to the taxonomic level of genus in the soil, and rhizosphere, roots, stem and grains of maize plants when weeds were removed manually (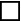), or plots were treated with glyphosate (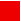) or paraquat (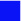) at the first sampling (soil), at flowering (rhizosphere and roots) or at harvest (stem and grains). The circles indicate the weeds were removed manually (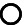), or plots were applied with glyphosate (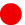) or paraquat (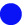), but they represented the second sampling, i.e., at flowering for the soil, at harvest (rhizosphere and roots) or after drying for 24 days (grains).

Overall, 35 different bacterial phyla were detected in the bulk soil, rhizosphere and maize plants (Supplementary Figure S3). Members of the Pseudomonadata dominated in the soil, rhizosphere and roots of maize plants, while Bacteroidota in the stem and grains. The most abundant bacterial genus in the bulk soil was *Priestia* (4.2%, mean of sampling at the onset and flowering), *Mucilaginibacter* in the rhizosphere (4.6%, mean of sampling at flowering and harvest) and *JC017* (*Marinilabiliaceae*, Bacteroidota) (30.6%, mean of sampling at flowering and harvest) in the roots (Figure 1A). Members of *JC017* dominated also in the stem (58.7%) and grains (73.4%), while an unassigned Alphaproteobacteria was the second most abundant bacterial group in the stem (35.8%) and grains (24.9%). Drying the grains for 24 days had little effect on the relative abundance of the bacterial groups.

**Figure 2:**
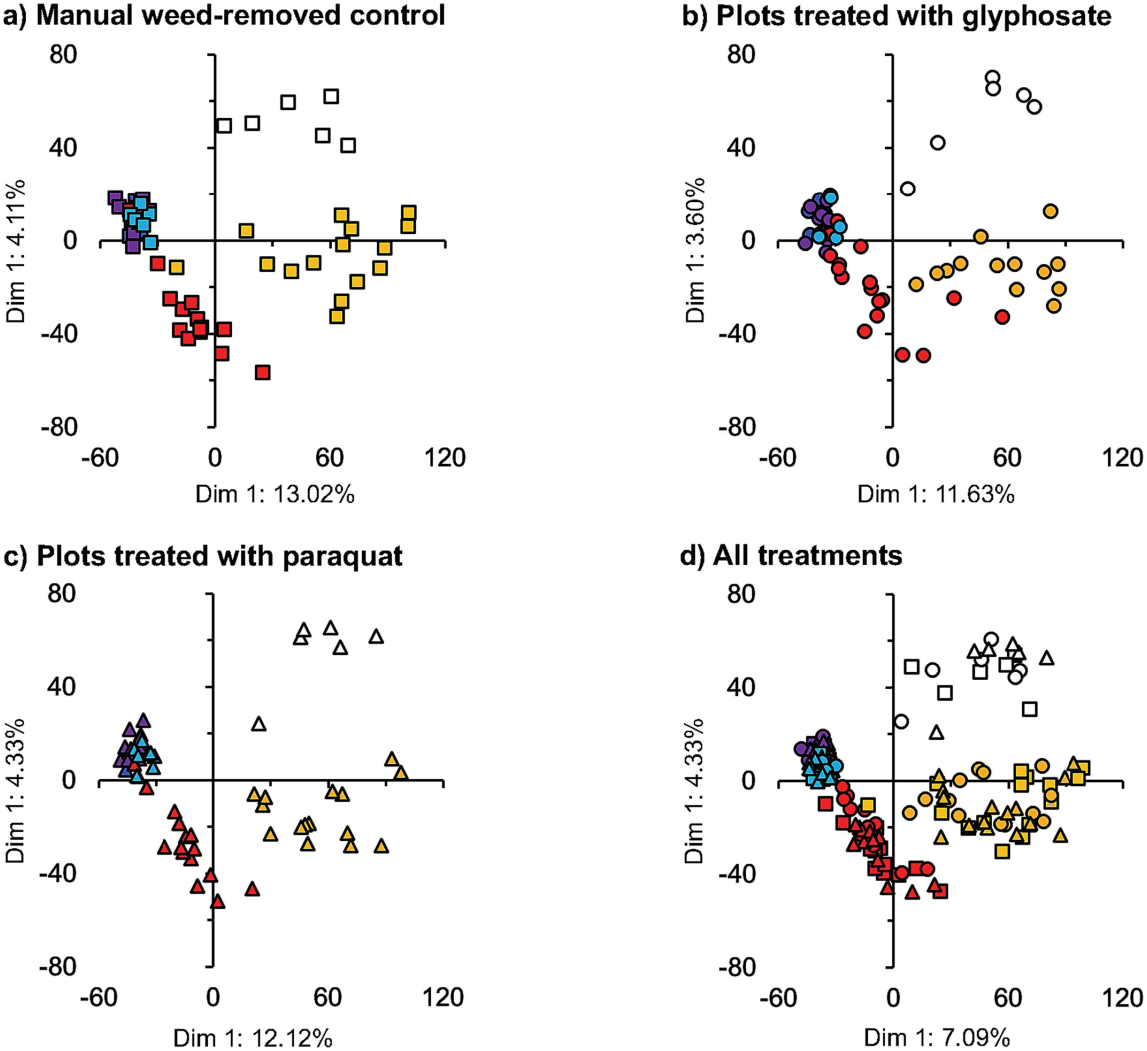
Principal component analysis (PCA) with all bacterial groups assigned up to the taxonomic level of genus **(a)** in the bulk soil (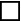), and rhizosphere (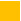), roots (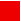), stem (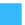) and grains (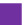) of maize plants (*Zea mays* L.) when weeds were removed manually, **(b)** in the bulk soil (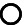), and rhizosphere (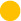), roots (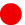), stem (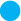) and grains (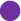) of maize plants when plots were treated with glyphosate, **(c)** in the bulk soil (△), and rhizosphere (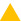), roots (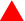), stem (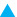) and grains (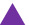) of maize plants when plots were treated with paraquat, and **(d)** all treatments.

The PCA did not separate the bacterial communities in the bulk soil, rhizosphere, or roots, stem or grains of the maize plants cultivated in plots treated with glyphosate or paraquat, or where weeds were removed manually (Figure 1B). Concordantly, the perMANOVA test indicated that the use of herbicides had no significant effect on the bacterial community structure in the bulk soil, rhizosphere, and roots, stem and fresh grains of the maize plants (Supplementary Table S2). The PCA, however, clearly separated the bacterial communities in the bulk soil sampled at the start of the experiment with those at flowering (Figure 1). The PCA also separated the bacterial communities in the bulk soil, rhizosphere, roots, and those in the grouped stem and grains from each other independent of the use of herbicides (Figure 2). The perMANOVA test indicated that the bacterial communities in the bulk soil, rhizosphere, roots and stem were significantly different from each other independent of the use of herbicides (*p* < 0.05) (Supplementary Table S3). However, the dispersion was also significant when comparing the bacterial communities in the soil vs. that in the rhizosphere at flowering in all treatments, and between the roots and rhizosphere and stem at harvest when plots were treated with paraquat (*p* < 0.05) (Supplementary Table S3). The bacterial community in the stem was not significantly different from that in the grains at harvest, and the dried grains and grains (fresh grains) at harvest.

Application of glyphosate or paraquat to the plots had a limited effect on the bacterial groups in the bulk soil, rhizosphere and the different plant parts compared to the plots where the weeds were removed manually (Supplementary Figure S4a). The effect size of the relative abundance of some bacterial groups was very large (≤ − 1.4 or ≥ 1.4) in the bulk soil of the glyphosate or paraquat treated plots compared to the manual weed-removed control plots, but none in the rhizosphere or the different plant parts. A similar result was found when comparing the changes in relative abundances of the bacterial groups over time with larger effects sizes found in the bulk soil, i.e., larger changes over time, than in the rhizosphere and roots (Supplementary Figure S5a). Interestingly the differences in the relative abundance of the bacterial groups between the plots as expressed by the effect size were similar as the effect of herbicide application on them (Supplementary Figure S5b). As such, the intrinsic variation in the relative abundances of the bacterial groups as evidenced by the effect sizes was similar as the effect of herbicide application or changes over time. The relative abundances of the bacterial groups in the bulk soil vs. the rhizosphere, in the rhizosphere vs. the roots and in the roots vs. the stem were not affected by herbicide application, and were often larger than the effect of the use of herbicides (Supplementary Figure S4b).

### 3.3 Putative metabolic functions

**Figure 3:**
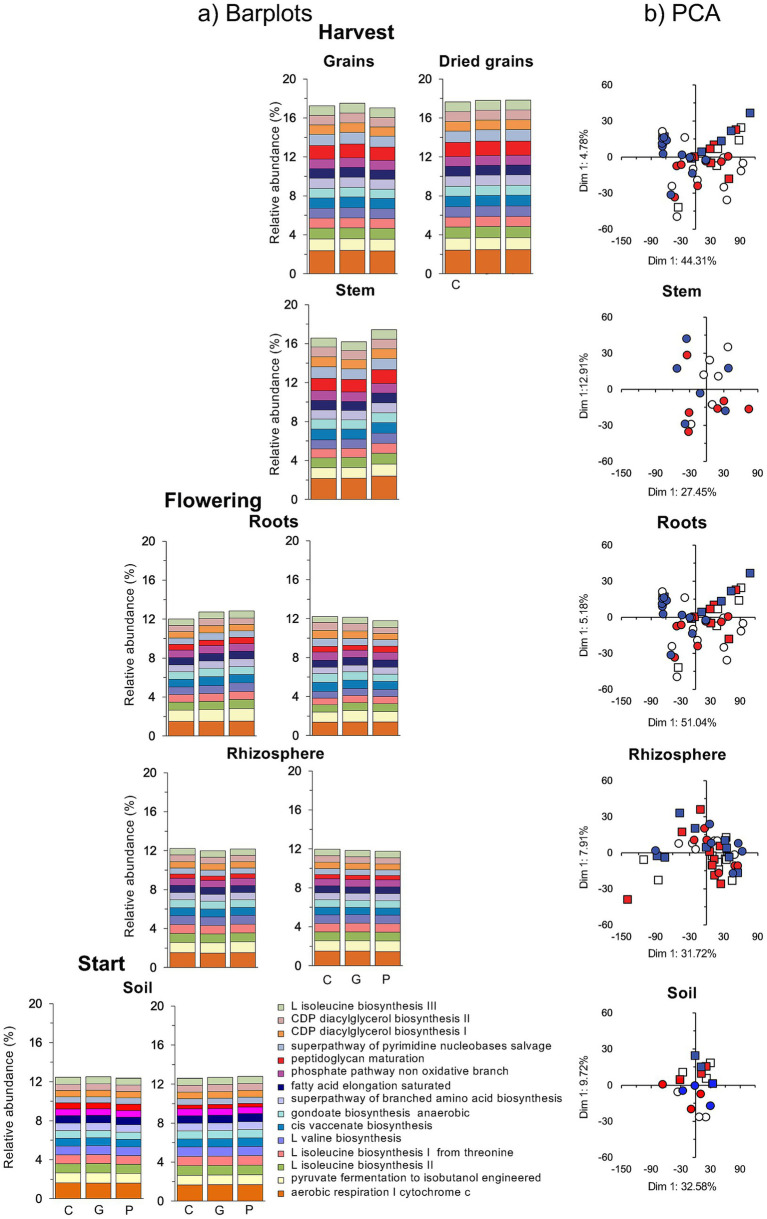
**(a)** Barplots with the relative abundance (%) of the 15 most abundant putative metabolic pathways in the soil, and rhizosphere, roots, stem and grains of maize plants (*Zea mays* L.) when weeds were removed manually (C) or plots were treated with glyphosate (G) or paraquat (P). **(b)** Principal component analysis (PCA) with all putative metabolic pathways in the soil, and rhizosphere, roots, stem and grains of maize plants. The legends used in the PCA are explained in the legends to Figure 2.

Aerobic respiration I cytochrome c (1.8%) was the most abundant putative metabolic pathway (Figure 3) and D0.helicase (1.1%) the most abundant EC (Data not shown). The PCA did not separate the bacterial putative metabolic structure in the bulk soil, rhizosphere, or roots, stem or grains of the maize plants cultivated in plots treated with glyphosate or paraquat, or where weeds were removed manually (Figure 3). The perMANOVA analysis indicated that herbicide application had no significant effect on the structure of the putative metabolic functions except in the dried grains (Supplementary Table S2). In the latter the structure of the putative metabolic functions was significantly different in the dry grains obtained from manual weed-removed control plots compared to those obtained from the plots treated with paraquat (perMANOVA test *p* < 0.05). Application of glyphosate or paraquat to the plots had a limited effect on the putative metabolic pathways of the bacterial groups in bulk soil, rhizosphere and the different plant parts compared to the manual weed-removed control ones (Figure 3).

**Figure 4:**
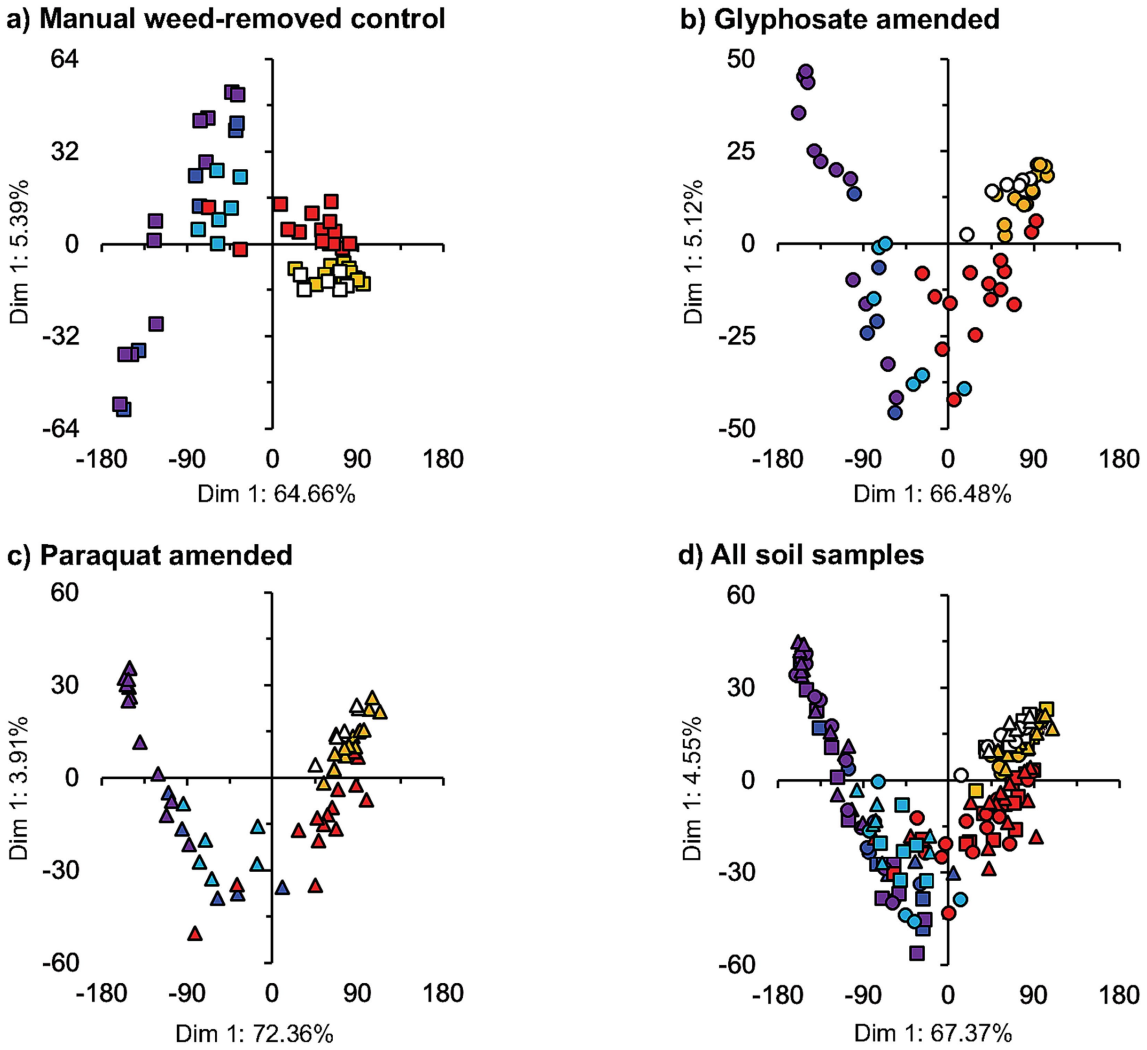
Principal component analysis (PCA) with the putative metabolic pathways **(a)** in the soil (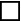), and rhizosphere (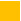), roots (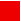), stem (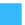) and grains (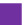) of maize plants (*Zea mays* L.) when weeds were removed manually, **(b)** in the soil (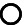), and rhizosphere (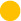), roots (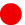), stem (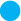) and grains (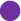) of maize plants when plots were treated with glyphosate and **(c)** in the soil (△), and rhizosphere (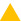), roots (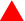), stem (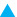) and grains (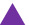) of maize plants when plots were treated with paraquat, and **(d)** all soil samples.

**Figure 5:**
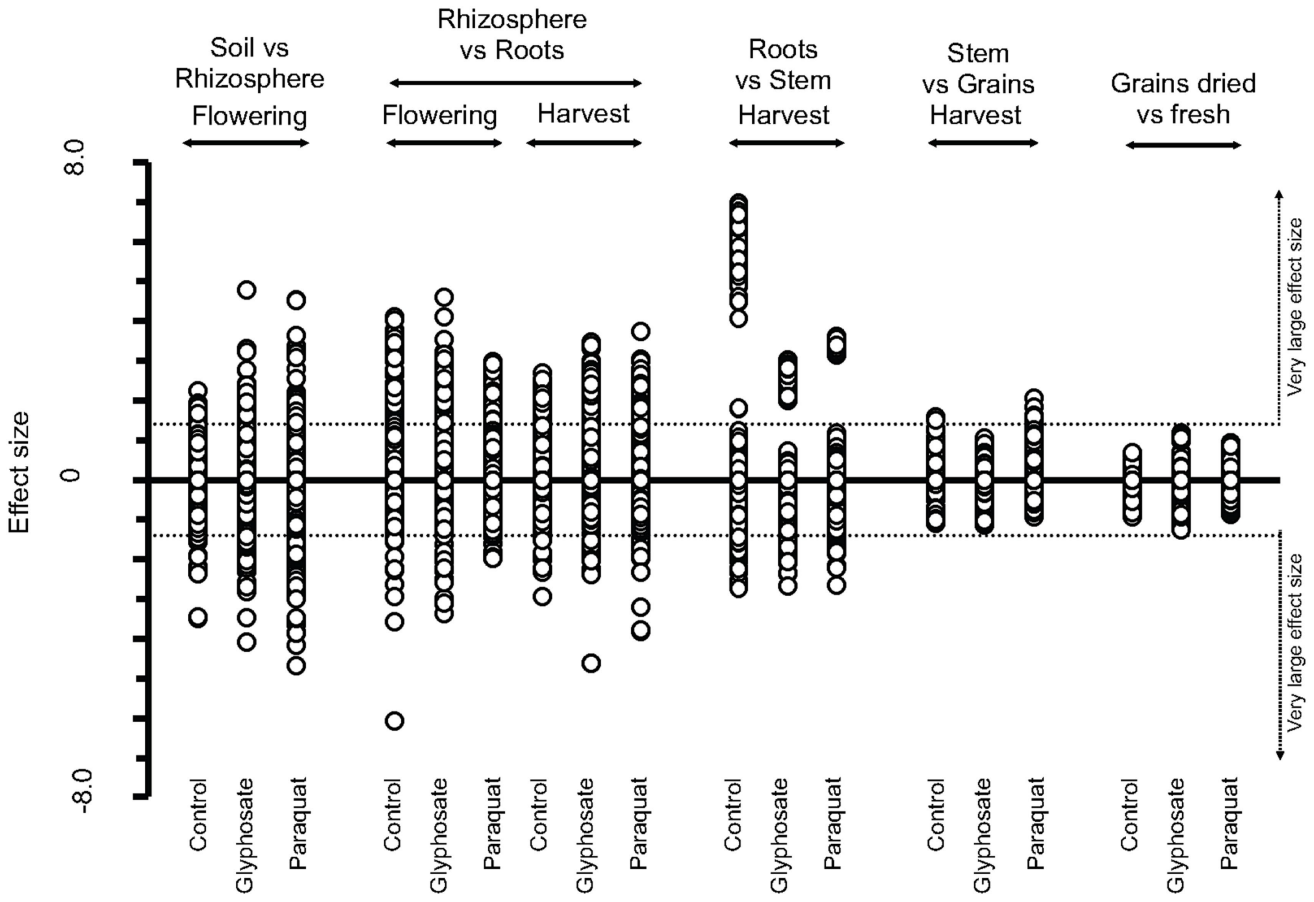
Effect sizes comparing the relative abundance of the putative metabolic functions in the soil vs. rhizosphere and rhizosphere vs. roots at flowering, rhizosphere vs. roots, roots vs. stem and stem vs. grains of maize (*Zea mays* L.) at harvest in the treatment where weeds were removed manually (control), or plots were treated with glyphosate or paraquat. The effect size, which is defined as the difference between groups divided by the maximum dispersion within group A or B, was calculated with the ALDEx2 package using the aldex.ttest argument (Gloor et al., 2023). A negative value indicates that the relative abundance of the putative metabolic functions was higher in the first mentioned compartment than in the second mentioned compartment and a positive value the opposite. Vertical lines indicate very large effect sizes (≤ − 1.4, ≥ 1.4).

The PCA separated clearly the putative metabolic pathways in the bulk soil and rhizosphere, from those in the roots and from those in the stem and grains although the replicated samples showed a large variation (Figure 4). The perMANOVA test indicated that the putative metabolic pathways in the bulk soil, rhizosphere, roots and stem were mostly significantly different from each other independent of the use of herbicides (*p* < 0.05, Supplementary Table S3). The putative metabolic pathways in the stem were not significantly different from those in the grains at harvest independent of the use of herbicides, and the dried grains and grains when weeds were removed manually. However, the dispersion was also significant when comparing the putative metabolic pathways between the rhizosphere and roots at flowering, and the dried grains and the grains at harvest when the plots werer treated with glyphosate or paraquat (*p* < 0.05). Additionally, the dispersion was also significant for the putative metabolic pathways between the rhizosphere and roots at flowering when the soil was treated with paraquat (*p* < 0.05). Interestingly, there was a distinctive group of putative metabolic pathways that was enriched in the stem of maize plants compared to their relative abundance in the roots more accentuated in the plants cultivated in the plots where the weeds were removed manually than when treated with herbicides (Figure 5, Supplementary Table S4).

## 4 Discussion

### 4.1 The effect of the herbicides on the bacterial community structure

At the onset of the experiment, i.e., before the herbicides were applied and the maize was planted, members of Pseudomonata (formerly Proteobacteria) were the most abundant in soil followed by Actinobacteriota, Bacillota (formerly Firmicutes) and Acidobacteriota. Agricultural practices, the type of crops cultivated, soil characteristics and climatical conditions will define which bacterial groups dominate (Gupta et al., 2022; Mo et al., 2024; Zong et al., 2024), but generally the same phyla are the most abundant in soil (Mhete et al., 2020). For instance, Duan et al. (2022) found that Pseudomonata, Acidobacteriota, Actinobacteriota were the dominant bacterial phyla in soils under long-term conservation management practices, and together with Verrucomicrobiota and Chloroflexota accounted for ∼70% of the total bacterial community in soil cultivated with cotton. Mhete et al. (2020) studied the bacterial community under different land-use regimes and found that five phyla in the order Pseudomonata > Actinobacteriota > Bacillota > Bacteroidota > Acidobacteriota dominated in all the soils studied.

In a previous study with soil from the same experimental site, the changes in the relative abundance of bacterial groups over the growing season were large and even within 7 days differences were noticeable (Romero-Salas et al., 2021). A similar pattern emerged in this study. The bacterial community in the bulk soil sampled at flowering was different from that at the start because of the agricultural practices applied. However, the inherent variation of the bacterial groups in the experimental plots, i.e., the random variation, must be considered. Comparing the bacterial groups in the different treatments before the herbicide was applied showed the random natural variation between the plots. As such, the changes in the bacterial community after sowing and herbicide application were partially due to the inherent variation in the plots before the herbicides were applied or in the manual weed-removed control plots, but not due to the application of the herbicide. It must be stressed, however, that the sampling took place in the second year of the experiment and, although highly unlikely, a possible remanent effect of the first application of the herbicides cannot be discarded and might have influenced the inherent variability.

A similar pattern emerged when considering the putative metabolic pathways. It should be stressed that this was the second year of the experiment with herbicides so some of the inherent variations between the plots might have been due to the application of herbicides the previous growing season. Follow-up studies in the following years might be necessary to confirm a possible effect of previous herbicide application on the bacterial communities in the next season.

Application of glyphosate or paraquat to the plots had no significant effect on the bacterial community structure and only a small effect on soil bacterial groups in the soil compared to the soil of the plots where the weeds were removed manually. There are different reasons for this. First, the amount of herbicides applied in the field experiment was low, i.e., 1,089 g glyphosate ha^−1^ and 400 g paraquat ha^−1^, and the herbicide was applied only once. The herbicides were applied early in this study which increases their efficiency and reduces the amount of herbicides that have to be applied to control the weeds and maximize the maize yields (Soltani et al., 2016; Sumekar et al., 2024). Second, the characteristics of the herbicides are such that they are quickly adsorbed onto the soil matrix or dissipated in water (Amondham et al., 2006; Okada et al., 2016). The Canadian Council of Ministers of the Environment (2012) reported that the bioconcentration factor of Roundup, a glyphosate formulation, was 1.6 in bluegill sunfish (*Lepomis macrochirus*), and ranged between 10.0 and 42.3 in carp (*Cyprinus carpio*) and 12.0 and 35.4 in tilapia (*Oreochromis mossambicus*). They stated that glyphosate has a low octanol–water partition coefficient (log K_ow_ = − 3.2 to −2.8) and highly soluble in water where it can quickly dissipate. The bioconcentration factor of paraquat ranges from 0.1 to 7.8 depending on the species studied (Katagi, 2010). Its octanol–water partition coefficient is low (log K_ow_ = − 4.5 at 20 °C) and highly soluble in water (620 g l^−1^ at 20 °C across pH range) where it quickly dissipates (FAO, 2006). As such, both herbicides remain mostly in the topsoil layer (0–5 cm) and their translocation to the plant rhizosphere will be limited (Amondham et al., 2006; Okada et al., 2016). Third, the soil was characterized by a high clay content (310 g kg^−1^) which will increase the fixation of the herbicides on the soil matrix and reduce their mobility (Amondham et al., 2006; Okada et al., 2016). Fourth, glyphosate can be translocated from the leaves to the roots and be excreted but had did have little or no effect on rhizosphere bacterial community in a study with transgenic Roundup Ready® soybean (Arango et al., 2014) or *EPSPS*-Transgenic Soybean Line ZUTS31 (Lu et al., 2018). In this study, glyphosate might have been translocated by the treated weeds from the leaves to the roots and excreted, but its effect on the bacterial community in the rhizosphere of the emerging maize plants was small. The translocation of paraquat in leaves depends on light and the resistance of the plant to paraquat (Soar et al., 2003), but its translocation from the leaves to the roots is considered low as it damages the plants immediately.

Only one bacterial genus in the glyphosate treated soil, i.e., *Haliangium_463188*, and one in the paraquat amended soil, i.e., *Flavobacterium*, showed a strong increase after the herbicide application. Glyphosate is degraded biologically via the aminomethylphosphonic acid (AMPA) and the sarcosine/glycine pathway in soil forming three intermediate products AMPA, sarcosine or glycine (Aslam et al., 2023). Two strains belonging to the myxobacterial genus *Haliangium* (*H. ochraceum and H. tepidum*) have been isolated from coastal regions and are differentiated from known terrestrial myxobacteria as they require some salt (2–3% NaCl) (Fudou et al., 2002). Other members of *Haliangium* (Myxococcota; Polyangia; Haliangiales; Kofleriaceae) are unclassified or environmental species (Schoch et al., 2020). Members of *Flavobacterium,* as many other bacteria, are known degraders of herbicides (Salam et al., 2026), such as glyphosate (e.g., Singh et al., 2020). Its capacity to degrade paraquat has not been described yet although different bacterial groups such as *Bacillus*, *Pseudomonas, Kocuria, Enterobacter, Chryseobacterium, Corynebacterium, Acinetobacter, Paenibacillus, Lerclercia* and *Proteus*, can use paraquat as sole C source (Ataikiru et al., 2020). As such, it would be no surprise if members of *Flavobacterium* could do the same. Little is known about the capacity of *Haliangium* to degrade glyphosate or that of *Flavobacterium* to degrade paraquat so further studies will be necessary to investigate if this enrichment of members of *Haliangium* and *Flavobacterium* was a random effect or if they were enriched by the herbicide application and could degrade it.

The relative abundance of *Pseudomonas* of which some members are known degraders of glyphosate [e.g., *Pseudomonas* _E_647464 (Zhang et al., 2024)] increased 5.1-times in soil of plots treated with the herbicide compared to the plots where the weeds were removed manually, i.e., at the start of the experiment, but that of *Bradyrhizobium*, which also includes glyphosate degraders, did not increase (Hernández-Guijarro et al., 2021). As such, not only the capacity to degrade glyphosate might enrich bacterial groups in soil treated with this herbicide but also soil characteristics.

Zhang et al. (2024) reported that the breaking of the C-N bond of glyphosate was primarily catalyzed by glycine oxidase in *Pseudomonas alcaligenes* Z1-1 (*thiO* gene) and not by glyphosate oxidoreductase. In this study the relative abundance of glycine oxidase as determined with EC or KO did not increase in the soil of the glyphosate treated plots. The relative abundance of other genes involved in the degradation of glyphosate *glpA*, *aroA*, *soxB* and *argA* (Zhang et al., 2024) did neither increase in this study. It must be stressed that these genes were not measured directly but were putative and derived from the bacterial groups detected in the soil. Shotgun metagenomics or primers for these specific genes might be necessary to confirm these results (Hernández-Guijarro et al., 2021).

### 4.2 Differential effects of glyphosate and paraquat on bacterial community in roots, stem and grains

The bacterial communities in the bulk soil, rhizosphere, roots and the different plant parts will be different due to different conditions, the effect of the plant, the soil–plant barrier and the limited mobility of bacteria within the plant. The bacterial community in the rhizosphere will change compared to the bulk soil as root exudates and dying roots provide easily decomposable organic material that heterotrophs metabolize providing C substrate for even more bacteria (McNear, 2013; Pantigoso et al., 2022; Chepsergon and Moleleki, 2023). Ling et al. (2022) analyzed 557 studies that compared the bacterial community in bulk versus rhizosphere soil and found that Bacteroidetes, Pseudomonadata, and other copiotrophs were enriched in the rhizosphere. In this study Bacteroidetes, Pseudomonadata and other copiotrophs, such as *Streptomyces*, were also enriched in the rhizosphere compared to the bulk soil. It has also been suggested that the endophytes of the grains do contribute to the rhizosphere bacterial community so their possible impact on the rhizosphere bacterial population cannot be excluded (Johnston-Monje et al., 2016). Although the bacterial communities in the rhizosphere were separated from those in the rhizosphere (PCA test) and although this was confirmed by the perMANOVA analysis, the dispersion test was also significant independent of how the weeds were treated, so one should be careful in interpreting these results. More studies will be required to confirm these results.

The bacterial community in the roots contains rhizosphere bacteria, i.e., those that can pass the root surface barrier, but also grain endophytes (Vandana et al., 2021; Hereira-Pacheco et al., 2021). Miranda-Carrazco et al. (2022) found that the alpha diversity based on Hill numbers was lower in the roots and stem of maize plants than in the rhizosphere as found in this study. The bacterial diversity in the roots was lower than in the rhizosphere as a limited number of bacteria will pass the root surface and the number of bacteria in the grains is low. Additionally, conditions within the roots are different from those in the rhizosphere as C substrate for growth is less diverse, and the O_2_ and nutrient content is lower than in the rhizosphere. Orole and Adejumo (2011) studied bacteria in two maize cultivars in Nigeria and isolated eight genera in both cultivars and a ninth in one cultivar. Most of the genera reported by them, e.g., *Azospirillum, Bacillus, Cellulomonas, Pseudomonas* and *Staphylococcus,* were detected in the roots in this study and if not then other members of the same families, e.g., Enterobacteriaceae, Micrococcaceae and Microbacteriaceae. Cun et al. (2022) studied the effect of soil type and maize variety on culturable endophytic bacterial and found that most isolates belonged to *Bacillus* (52.30%), *Streptomyces* (13.22%), *Paenibacillus* (6.32%) and *Pseudomonas* (4.60%), all of them detected in the roots of maize investigated in this study.

The bacterial community in the stem was a further subset of the root bacterial community as the stem forms a further barrier from the roots. Only 135 bacterial groups were detected in the stem up to the taxonomic level of genus compared to 552 in the roots with three that formed > 99% of the bacterial community, i.e., *Marinilabiliaceae* bacterium JC017 (NCBI:txid2234116) that belongs to Bacteroidota, an undefined phylotype belonging to the Alphaproteobacteria and *Phytoplasma* a member of the *Acholeplasmataceae* belonging to the Bacillota. Phylotypes belonging to *Phytoplasma* cause maize bushy stunt phytoplasma (MBSP) in maize (*Zea mays* L.) across Latin America (Orlovskis et al., 2017). They are phytopathogenic bacteria also of native maize plants and obligate parasites of plant phloem and vectored by leafhoppers in the genus *Dalbulus* (Hemiptera: Cicadellidae) at high elevations in Southeast Mexico (Pérez-López et al., 2018). *Marinilabiliaceae* have been found as nitrogen fixing bacteria in marine sediments (Yu et al., 2023). Although the bacterial communities in the roots were separated from those in the stem (PCA test) and although this was confirmed by the perMANOVA analysis, the dispersion test was also significant in plots where weeds were treated with paraquat so one should be careful in interpreting these results. More studies will be required to confirm a possible effect of paraquat on the dispersion of the bacterial communities in the stem and roots.

Acuña et al. (2023) studied the bacterial communities in ungerminated and germinated grains of commercial vegetables, i.e., parsley, carrot, cabbage, broccoli and tomato, and found that Pseudomonadota and Bacillota dominated in both. The same bacterial phyla dominated in the sampled maize grains. The bacterial community in the grains was similar to that in the stem and if a barrier existed its effect was limited. Although 109 different bacterial groups up to the taxonomic level of genus were detected in the grains, only two dominated, *JC017* and an undefined member of the Alphaproteobacteria, which represented > 99% of the total relative abundance. Interestingly the relative abundance of *JC017* and an undefined member of the Alphaproteobacteria showed a constant increase from the soil upwards to the maize grains, i.e., from < 1% in the soil to > 99% in the grains.

In this study the bacterial diversity considering the bacterial groups assigned up to the taxonomic level of genus was larger in the rhizosphere than in the bulk soil, although the opposite has been reported (Ling et al., 2022; Navarro-Noya et al., 2022) or no differences were detected (Afanador-Barajas et al., 2021). As such, climatical conditions, e.g., precipitation, soil characteristics, e.g., pH, and type of plant and its growth stage will control the bacterial diversity in soils (Tan et al., 2020) and the rhizosphere (e.g., Schneijderberg et al., 2020; Wan et al., 2020), and differences between the endophytic community in the roots, stem and grains of plants.

### 4.3 Putative metabolic functions

The putative metabolic functions were not affected by the application of the herbicides in the different compartments studied except in the dried grains. In the latter, the application of paraquat had a significant effect on the putative metabolic functions compared to the plots where the weeds were removed manually. It is difficult to explain why this might have happened as the application of paraquat occurred before the maize plants emerged, but as the effect is highly significant it is important to repeat the experiment to confirm this finding using shotgun metagenomics.

The putative metabolic structure of the pathways, KO and EC in the soil, rhizosphere, roots, stem and grains were separated as visualized by PCA, but some differences were detected when compared to the bacterial community structures. The bacterial community was clearly distinct in the soil and rhizosphere, but the separation was not always that clear when considering the putative metabolic pathways. The putative metabolic functions showed a large variation in the grains, which was absent when considering the bacterial groups. Additionally, there was a larger difference in the structure of the putative metabolic functions in the stem versus the roots than when considering the bacterial groups. A limited number of putative metabolic functions showed a larger increase in the stem compared to the roots (effect size at least > 2.0 in each of the treatments) than that of other functions. The decreases in those putative functions in the stem compared to the roots were often larger. Most of the putative pathways that were enriched in the stem compared to the roots were related to biosynthesis, while those that showed a decrease were related to degradation. In the stem, bacterial metabolism is more anabolic in terms of biosynthesis of N biomolecules (amino acids and nucleotides) and the energy pathways to support them compared to the roots. Contrary, the metabolic profile in the roots was more oriented to catabolism of organic molecules. This could be explained by a higher bioavailability of a wider range of biomolecules in the roots than in stem where the bacterial metabolic capabilities are more oriented towards synthesis of their own metabolites needed for growth.

In previous experiments, we found that changes in the relative abundance of putative metabolic functions were smaller than those of the bacterial groups (Zarco-González et al., 2023). Xia et al. (2022) studied the assembly of functional genes and taxa in soil and the gut of the earthworm under vanadium stress and found that the taxa were more sensitive to stress in soil compared with functional genes, but not in the earthworm gut. This suggested the existence of bacterial functional redundancy in soil, but not in the earthworm gut. Miralles et al. (2021) studied the functional and taxonomic effects of organic amendments on the restoration of semiarid quarry soil. They reported that both taxonomic and functional metagenomic sequencing profiling separated clearly the organic amended soil from the unamended control samples. They also stated that although the taxonomic differences were clear, the functional redundancy was higher than expected.

Results based on putative metabolic functions could be doubted as they were based on indirect assignations. The question could be asked if the lower variations in the relative abundance of putative metabolic functions than those of the bacterial groups was a “real” phenomenon or an artefact. However, variations in bacterial genes in composted cow manure determined by shotgun metagenomics were also smaller than those of bacterial groups (Romero-Yahuitl et al., 2024). Jiao et al. (2019) found that functional profiles showed greater resilience than taxonomic ones in an arable soil amended with inorganic and organic contaminants (phenanthrene, *n*-octadecane, and CdCl_2_) cultivated with three common legume plants based on shotgun metagenomics. This suggest that changes in bacterial groups were more stochastic than those of the metabolic functions, i.e., bacterial groups can perform the same metabolic function and as such there was a clear functional redundancy (Louca et al., 2018). Additionally, variations in the putative metabolic pathways were also much lower than those of the bacterial groups in the bulk soil, rhizosphere and maize plants so that the effect sizes comparing the bacterial groups in the different parts studied were often larger than those of the bacterial groups.

## 5 Conclusion

Application of the herbicides glyphosate and paraquat to soil before the emergence of maize plants did not affect the bacterial community structure in the bulk soil, rhizosphere, roots, stem and grains plants at flowering and/or harvest. The relative abundance of some bacterial groups and their putative metabolic functions changed in soil after herbicide application. However, the magnitude of the “effect” was similar to the difference detected between the plots and before the herbicides were applied or in the manual weed-removed control plots. *Haliangium_463188* was enriched strongly in the glyphosate and a member of *Flavobacterium* in the paraquat-amended soil, but their possible participation in the removal of the herbicides requires further studies.

The bacterial community structures were different between the bulk soil, rhizosphere, roots and stem and grains of the maize plants with large changes in the relative abundance of the bacterial groups and two phylotypes, i.e., an unidentified Alphaproteobacteria and uncultured member of the *Marinilabiliaceae* (*JC017*, Bacteroidota), dominating in the stem and grains of the maize plants. The variations in putative metabolic in the bulk soil, rhizosphere and the maize plants were less accentuated although they were clearly different between the bulk soil and rhizosphere, roots, and stem and grains. Most of the putative metabolic pathways that were enriched in the stem compared to the roots were related to biosynthesis, while those that showed a decrease were related to degradation. Shotgun metagenomics or mRNA studies will be required to answer some of the questions raised by this study.

## Data Availability

The datasets presented in this study can be found in online repositories. The names of the repository/repositories and accession number(s) can be found at: https://www.ncbi.nlm.nih.gov/, PRJNA1191893.

## Supplementary material

SUPPLEMENTARY Figure S1 Experimental design, and soil and maize plant sampling.

SUPPLEMENTARY Figure S2 Hill numbers at *q* = **(a)** 0, **(b)** 1, and **(c)** 2 of the bacterial groups up to the taxonomic level of genus in the bulk soil, rhizosphere, roots, stem and grains of maize plants (*Zea mais* L.) when weeds were removed manually (C), or plots were treated with glyphosate (G) or paraquat (P) at the start of the experiment, flowering and harvest. ND: Could not be included in the non-parametric statistical analysis.

SUPPLEMENTARY Figure S3 Barplots with the relative abundance (%) of the 15 most abundant bacterial phyla in the soil, and rhizosphere, roots, stem and grains of maize plants when weeds were removed manually (C), or plots were treated with glyphosate (G) or paraquat (P).

SUPPLEMENTARY Figure S4 Effect sizes obtained when **(a)** comparing the relative abundance of bacterial groups up to the taxonomic level of genus in the soil of the treatment where weeds were removed manually (C) vs. those treated with glyphosate (G), or vs. those treated by paraquat (P), or rhizosphere, roots, stem or grains of maize (*Zea mays* L.) at the start of the experiment, flowering or harvest and **(b)** in the soil vs. rhizosphere and rhizosphere vs. roots at flowering, rhizosphere vs. roots, roots vs. stem and stem vs. grains of maize (*Zea mays* L.) at harvest in the treatment where weeds were removed manually (ontrol), or plots were treated with glyphosate or paraquat. The effect size is explained in the legends to Figure 5. A negative value indicates that the relative abundance of the bacterial group was higher in the first mentioned treatment or compartment than in the second and a positive value the opposite. Vertical lines indicate very large effect sizes (≤ − 1.4, ≥ 1.4).

SUPPLEMENTARY Figure S5 Effect sizes when **(a)** comparing the relative abundance of bacterial groups up to the taxonomic level of genus in the first sampling vs. the second sampling in the bulk soil, rhizosphere and roots of maize when weeds were removed manually (Control), or plots treated with glyphosate or paraquat, or rhizosphere, roots, stem or grains of maize (*Zea mays* L.) at the start of the experiment, flowering or harvest and **(b)** comparing plot 1 vs. plot 2, plot 1 vs. plot 3, and plot 2 vs. plot 3 in the bulk soil, rhizosphere, roots, stem and grains of maize (*Zea mays* L.) at the start of the experiment, flowering and harvest. The effect size is explained in the legends to Figure 5. A negative value indicates that the relative abundance of the bacterial group was higher in the first mentioned sampling or plot than in the second and a positive value the opposite. Vertical lines indicate very large effect sizes (≤ − 1.4, ≥ 1.4).
