# Supplementary material for: Application of glyphosate or paraquat before the emergence of maize plants (Zea mays L.) had no detectable effect on the rhizosphere or endophyte bacterial community structure under the tested field conditions
Source: Front Microbiol. 2026 Aug 5;17:1892064. doi: 10.3389/fmicb.2026.1892064 (PMC13485912; doi:10.3389/fmicb.2026.1892064)
Supplement: Supplementary file 1 [file Table_1.DOCX]

**Table S1** Effect of herbicide application on the Hill numbers of bacterial groups assigned up to the taxonomic level of genus at *q* = 0, 1 and 2 in the bulk soil, rhizosphere, roots, stem and grains.

| —————————————————————————————————— | | | | | | | | | |
| --- | --- | --- | --- | --- | --- | --- | --- | --- | --- |
|  |  |  | *q* = 0 | | *q* = 1 | | | *q* = 2 | |
|  |  |  | —————— | | —————— | | | —————— | |
| Sampled | Time |  | F value | *p* value | | F value | *p* value | F value | *p* value |
| —————————————————————————————————— | | | | | | | | | |
| Bulk soil | Start |  | 0.105 | 0.903 | | 0.016 | 0.984 | 0.742 | 0.547 |
|  | Flowering | | 0.353 | 0.723 | | 0.368 | 0.714 | 0.764 | 0.525 |
| Rhizosphere | Flowering | | 0.376 | 0.715 | | 0.083 | 0.922 | 0.102 | 0.906 |
|  | Harvest | | 0.651 | 0.572 | | 0.232 | 0.806 | ND ^a^ | ND |
| Roots | Flowering | | 0.383 | 0.706 | | 1.291 | 0.378 | 3.196 | 0.163 |
|  | Harvest | | 0.167 | 0.852 | | 0.042 | 0.959 | 0.021 | 0.980 |
| Stem | Harvest | | 0.706 | 0.547 | | ND | ND | ND | ND |
| Grains | Harvest | | 0.144 | 0.871 | | ND | ND | ND | ND |
|  | Dried | | 3.171 | 0.177 | | ND | ND | ND | ND |
| —————————————————————————————————— | | | | | | | | | |
| ^a^ ND: Not determined. | | | | | | | | | |
| —————————————————————————————————— | | | | | | | | | |
