# Supplementary material for: Application of glyphosate or paraquat before the emergence of maize plants (Zea mays L.) had no detectable effect on the rhizosphere or endophyte bacterial community structure under the tested field conditions
Source: Front Microbiol. 2026 Aug 5;17:1892064. doi: 10.3389/fmicb.2026.1892064 (PMC13485912; doi:10.3389/fmicb.2026.1892064)
Supplement: Supplementary file 2 [file Table_2.DOCX]

| **Table S2.** Permutational multivariate analysis of variance (perMANOVA) to determine the effect of herbicide application, i.e. the plots where the weeds were removed manually (Control), or treated with glyphosate or paraquat, on the bacterial groups assigned up to the taxonomic level of genus and the putative metabolic pathways in the soil, rhizosphere, roots, stem or grains. Permutation multivariate analysis of dispersion (PERMDISP) was used to determine if the dispersion within the compared treatments was significant when the perMANOVA test detected a significant effect.  ⎯⎯⎯⎯⎯⎯⎯⎯⎯⎯⎯⎯⎯⎯⎯⎯⎯⎯⎯⎯⎯⎯⎯⎯⎯⎯⎯⎯⎯⎯⎯⎯⎯⎯⎯ | | | | | | | | | | | | |  |
| --- | --- | --- | --- | --- | --- | --- | --- | --- | --- | --- | --- | --- | --- |
| Plant part | | r2 | F value | | *p* value | | | F value | | *p* value | |  |  |
| ⎯⎯⎯⎯⎯⎯⎯⎯⎯⎯⎯⎯⎯⎯⎯⎯⎯⎯⎯⎯⎯⎯⎯⎯⎯⎯⎯⎯⎯⎯⎯⎯⎯⎯⎯ | | | | | | | | | | | | |  |
| Bacterial groups up to the taxonomic level of genus | | | | | | | | | | | | |  |
| ⎯⎯⎯⎯⎯⎯⎯⎯⎯⎯⎯⎯⎯⎯⎯⎯⎯⎯⎯⎯⎯⎯⎯⎯⎯⎯⎯⎯⎯⎯⎯⎯⎯⎯⎯ | | | | | | | | | | | | |  |
| All | |  |  | | |  | | |  | |  | |  |
| Soil start | | 0.235 | 0.92 | | | 0.942 | | |  | |  | |  |
| Soil flowering | | 0.235 | 0.92 | | | 0.898 | | |  | |  | |  |
| Rhizosphere flowering | | 0.078 | 1.02 | | | 0.310 | | |  | |  | |  |
| Rhizosphere harvest | | 0.109 | 0.92 | | | 0.883 | | |  | |  | |  |
| Roots flowering | | 0.080 | 1.04 | | | 0.185 | | |  | |  | |  |
| Roots harvest | | 0.119 | 1.01 | | | 0.336 | | |  | |  | |  |
| Stem harvest | | 0.118 | 1.00 | | | 0.459 | | |  | |  | |  |
| Fresh grains harvest | | 0.119 | 0.94 | | | 0.976 | | |  | |  | |  |
| Grains dried for 24 days | | 0.057 | 0.98 | | | 0.791 | | |  | |  | |  |
| ⎯⎯⎯⎯⎯⎯⎯⎯⎯⎯⎯⎯⎯⎯⎯⎯⎯⎯⎯⎯⎯⎯⎯⎯⎯⎯⎯⎯⎯⎯⎯⎯⎯⎯⎯ | | | | | | | | | | | | |  |
| Putative metabolic pathways | | | | | | | | | | | | |  |
| ⎯⎯⎯⎯⎯⎯⎯⎯⎯⎯⎯⎯⎯⎯⎯⎯⎯⎯⎯⎯⎯⎯⎯⎯⎯⎯⎯⎯⎯⎯⎯⎯⎯⎯⎯ | | | | | | | | | | | | |  |
| All | | 0.007 | | 0.63 | | | 0.630 | |  | |  | |  |
| Soil start | | 0.230 | | 0.90 | | | 0.605 | |  | |  | |  |
| Soil flowering | | 0.229 | | 0.89 | | | 0.548 | |  | |  | |  |
| Rhizosphere flowering | | 0.058 | | 0.74 | | | 0.903 | |  | |  | |  |
| Rhizosphere harvest | | 0.082 | | 0.67 | | | 0.843 | |  | |  | |  |
| Roots flowering | | 0.058 | | 0.65 | | | 0.734 | |  | |  | |  |
| Roots harvest | | 0.106 | | 1.07 | | | 0.364 | |  | |  | |  |
| Stem harvest | | 0.103 | | 0.87 | | | 0.679 | |  | |  | |  |
| Fresh grains harvest | | 0.068 | | 0.55 | | | 0.959 | |  | |  | |  |
| Grains dried for 24 days | | 0.137 | | 2.55 | | | 0.009* | |  | |  | |  |
|  | Control ^b^ vs Glyphosate | 0.081 | | 1.86 | | | 0.088 | | 0.147 | | 0.708 | |  |
|  | Control vs Paraquat | 0.175 | | 4.46 | | | 0.003** | | 0.315 | | 0.560 | |  |
|  | Glyphosate vs Paraquat | 0.057 | | 1.34 | | | 0.118 | | 0.822 | | 0.367 | |  |
| ⎯⎯⎯⎯⎯⎯⎯⎯⎯⎯⎯⎯⎯⎯⎯⎯⎯⎯⎯⎯⎯⎯⎯⎯⎯⎯⎯⎯⎯⎯⎯⎯⎯⎯⎯ | | | | | | | | | | | | | |

^a^ * Significant at *p* < 0.05, ^b^ Control: weeds were removed manually from the plots.

⎯⎯⎯⎯⎯⎯⎯⎯⎯⎯⎯⎯⎯⎯⎯⎯⎯⎯⎯⎯⎯⎯⎯⎯⎯⎯⎯⎯⎯⎯⎯⎯⎯⎯⎯
