# Supplementary material for: Application of glyphosate or paraquat before the emergence of maize plants (Zea mays L.) had no detectable effect on the rhizosphere or endophyte bacterial community structure under the tested field conditions
Source: Front Microbiol. 2026 Aug 5;17:1892064. doi: 10.3389/fmicb.2026.1892064 (PMC13485912; doi:10.3389/fmicb.2026.1892064)
Supplement: Supplementary file 3 [file Table_3.DOCX]

**Table S3.** Permutational multivariate analysis of variance (perMANOVA) to determine the effect of the cultivated maize plants, i.e. bulk soil, rhizosphere, roots, stem and grains on the bacterial groups assigned up to the taxonomic level of genus and the putative metabolic pathways in the plots where the weeds were removed manually (Control), or treated with glyphosate or paraquat. Permutation multivariate analysis of dispersion (PERMDISP) was used to determine if the dispersion within the compared treatments was significant when the perMANOVA test detected a significant effect.

| ⎯⎯⎯⎯⎯⎯⎯⎯⎯⎯⎯⎯⎯⎯⎯⎯⎯⎯⎯⎯⎯⎯⎯⎯⎯⎯⎯⎯⎯⎯⎯⎯⎯⎯⎯⎯⎯⎯⎯⎯ | | | | | | | |
| --- | --- | --- | --- | --- | --- | --- | --- |
|  | | perMANOVA | | | | PERMDISP ^a^ | |
|  | | ⎯⎯⎯⎯⎯⎯⎯⎯⎯⎯ | | | | ⎯⎯⎯⎯⎯⎯⎯ | |
|  |  | R^2^ | F value | | *P* value | F value | *P* value |
| ⎯⎯⎯⎯⎯⎯⎯⎯⎯⎯⎯⎯⎯⎯⎯⎯⎯⎯⎯⎯⎯⎯⎯⎯⎯⎯⎯⎯⎯⎯⎯⎯⎯⎯⎯⎯⎯⎯⎯⎯ | | | | | | | |
| Bacterial groups up to the taxonomic level of genus | | | | | | | |
| ⎯⎯⎯⎯⎯⎯⎯⎯⎯⎯⎯⎯⎯⎯⎯⎯⎯⎯⎯⎯⎯⎯⎯⎯⎯⎯⎯⎯⎯⎯⎯⎯⎯⎯⎯⎯⎯⎯⎯⎯ | | | | | | | |
| All treatments | All locations | 0.143 | 5.76 | | 0.001*** ^b^ |  |  |
| ⎯⎯⎯⎯⎯⎯⎯⎯⎯⎯⎯⎯⎯⎯⎯⎯⎯⎯⎯⎯⎯⎯⎯⎯⎯⎯⎯⎯⎯⎯⎯⎯⎯⎯⎯⎯⎯⎯⎯⎯ | | | | | | | |
| Control | All | 0.245 | 2.03 | | 0.001*** |  |  |
|  | Soil vs rhizosphere: flowering | 0.116 | 1.31 | | 0.041* | 10.27 | 0.011* |
|  | Rhizosphere vs roots: flowering | 0.140 | 2.61 | | 0.001*** | 3.58 | 0.069 |
|  | Rhizosphere vs roots: harvest | 0.156 | 1.85 | | 0.019* | 2.37 | 0.162 |
|  | Roots vs stem: harvest | 0.122 | 1.39 | | 0.007** | 1.43 | 0.272 |
|  | Stem vs FG ^c^: harvest | 0.090 | 0.99 | | 0.562 |  |  |
|  | FG vs DG ^d^: harvest | 0.059 | 0.94 | | 0.948 |  |  |
| ⎯⎯⎯⎯⎯⎯⎯⎯⎯⎯⎯⎯⎯⎯⎯⎯⎯⎯⎯⎯⎯⎯⎯⎯⎯⎯⎯⎯⎯⎯⎯⎯⎯⎯⎯⎯⎯⎯⎯⎯ | | | | | | | |
| Glyphosate | All | 0.232 | 1.93 | | 0.001*** |  |  |
|  | Soil vs rhizosphere: flowering | 0.121 | 1.38 | | 0.020* | 6.85 | 0.026* |
|  | Rhizosphere vs roots: flowering | 0.139 | 2.59 | | 0.001*** | 4.14 | 0.059 |
|  | Rhizosphere vs roots: harvest | 0.162 | 1.93 | | 0.005** | 2.74 | 0.129 |
|  | Roots vs stem: harvest | 0.108 | 1.21 | | 0.009** | 0.15 | 0.707 |
|  | Stem vs FG: harvest | 0.092 | 1.01 | | 0.464 |  |  |
|  | FG vs DG: harvest | 0.059 | 1.01 | | 0.406 |  |  |
| ⎯⎯⎯⎯⎯⎯⎯⎯⎯⎯⎯⎯⎯⎯⎯⎯⎯⎯⎯⎯⎯⎯⎯⎯⎯⎯⎯⎯⎯⎯⎯⎯⎯⎯⎯⎯⎯⎯⎯⎯ | | | | | | | |
| Paraquat | All | 0.248 | | 2.11 | 0.001*** |  |  |
|  | Soil vs rhizosphere: flowering | 0.138 | | 1.60 | 0.020* | 7.68 | 0.020* |
|  | Rhizosphere vs roots: flowering | 0.131 | | 2.42 | 0.001*** | 3.76 | 0.070 |
|  | Rhizosphere vs roots: harvest | 0.159 | | 1.90 | 0.005** | 9.15 | 0.011* |
|  | Roots vs stem: harvest | 0.125 | | 1.43 | 0.019* | 5.22 | 0.037* |
|  | Stem vs FG: harvest | 0.091 | | 1.00 | 0.471 |  |  |
|  | FG vs DG: harvest | 0.057 | | 0.97 | 0.787 |  |  |
| ⎯⎯⎯⎯⎯⎯⎯⎯⎯⎯⎯⎯⎯⎯⎯⎯⎯⎯⎯⎯⎯⎯⎯⎯⎯⎯⎯⎯⎯⎯⎯⎯⎯⎯⎯⎯⎯⎯⎯⎯ | | | | | | | |

**Table S3.** Continued.

| ⎯⎯⎯⎯⎯⎯⎯⎯⎯⎯⎯⎯⎯⎯⎯⎯⎯⎯⎯⎯⎯⎯⎯⎯⎯⎯⎯⎯⎯⎯⎯⎯⎯⎯⎯⎯⎯⎯⎯⎯ | | | | | | |
| --- | --- | --- | --- | --- | --- | --- |
| Putative metabolic pathways | | | | | | |
| ⎯⎯⎯⎯⎯⎯⎯⎯⎯⎯⎯⎯⎯⎯⎯⎯⎯⎯⎯⎯⎯⎯⎯⎯⎯⎯⎯⎯⎯⎯⎯⎯⎯⎯⎯⎯⎯⎯⎯⎯ | | | | | | |
| Control | All | 0.605 | 9.59 | 0.001*** |  |  |
|  | Soil vs rhizosphere: flowering | 0.166 | 1.98 | 0.076 |  |  |
|  | Rhizosphere vs roots: flowering | 0.233 | 4.55 | 0.001*** | 3.53 | 0.067 |
|  | Rhizosphere vs roots: harvest | 0.227 | 3.24 | 0.018* | 0.28 | 0.685 |
|  | Roots vs stem: harvest | 0.505 | 11.24 | 0.006** | 0.82 | 0.367 |
|  | Stem vs FG: harvest | 0.124 | 1.42 | 0.229 |  |  |
|  | FG vs DG: harvest | 0.043 | 0.67 | 0.628 |  |  |
| ⎯⎯⎯⎯⎯⎯⎯⎯⎯⎯⎯⎯⎯⎯⎯⎯⎯⎯⎯⎯⎯⎯⎯⎯⎯⎯⎯⎯⎯⎯⎯⎯⎯⎯⎯⎯⎯⎯⎯⎯ | | | | | | |
| Glyphosate | All | 0.668 | 12.85 | 0.001*** |  |  |
|  | Soil vs rhizosphere: flowering | 0.308 | 4.56 | 0.020* | 0.71 | 0.446 |
|  | Rhizosphere vs roots: flowering | 0.347 | 8.50 | 0.001*** | 9.36 | 0.006** |
|  | Rhizosphere vs roots: harvest | 0.268 | 3.66 | 0.061 |  |  |
|  | Roots vs stem: harvest | 0.272 | 3.73 | 0.019* | 0.03 | 0.886 |
|  | Stem vs FG: harvest | 0.128 | 1.46 | 0.169 |  |  |
|  | FG vs DG: harvest | 0.172 | 3.32 | 0.012** | 5.14 | 0.035* |
| ⎯⎯⎯⎯⎯⎯⎯⎯⎯⎯⎯⎯⎯⎯⎯⎯⎯⎯⎯⎯⎯⎯⎯⎯⎯⎯⎯⎯⎯⎯⎯⎯⎯⎯⎯⎯⎯⎯⎯⎯ | | | | | | |
| Paraquat | All | 0.704 | 15.17 | 0.001*** |  |  |
|  | Soil vs rhizosphere: flowering | 0.237 | 3.10 | 0.016* | 0.42 | 0.574 |
|  | Rhizosphere vs roots: flowering | 0.234 | 4.27 | 0.003** | 3.09 | 0.050* |
|  | Rhizosphere vs roots: harvest | 0.171 | 2.47 | 0.047* | 2.94 | 0.114 |
|  | Roots vs stem: harvest | 0.477 | 10.93 | 0.009** | 0.01 | 0.965 |
|  | Stem vs FG: harvest | 0.100 | 1.11 | 0.272 |  |  |
|  | FG vs DG: harvest | 0.221 | 4.53 | 0.010* | 1.78 | 0.183 |
| ⎯⎯⎯⎯⎯⎯⎯⎯⎯⎯⎯⎯⎯⎯⎯⎯⎯⎯⎯⎯⎯⎯⎯⎯⎯⎯⎯⎯⎯⎯⎯⎯⎯⎯⎯⎯⎯⎯⎯⎯ | | | | | | |

^a^ Permutation test for homogeneity of multivariate dispersions (betadisper argument, vegan, version 2.6-8), ^b^ * ≤ 0.05 and > 0.01, ** ≤ 0.01 and > 0.001, *** ≤ 0.001, ^c^ FG: Fresh grains, ^d^ DG: Dry grains.

⎯⎯⎯⎯⎯⎯⎯⎯⎯⎯⎯⎯⎯⎯⎯⎯⎯⎯⎯⎯⎯⎯⎯⎯⎯⎯⎯⎯⎯⎯⎯⎯⎯⎯⎯⎯⎯⎯⎯⎯
