# Supplementary material for: Application of glyphosate or paraquat before the emergence of maize plants (Zea mays L.) had no detectable effect on the rhizosphere or endophyte bacterial community structure under the tested field conditions
Source: Front Microbiol. 2026 Aug 5;17:1892064. doi: 10.3389/fmicb.2026.1892064 (PMC13485912; doi:10.3389/fmicb.2026.1892064)
Supplement: Supplementary file 4 [file Table_4.DOCX]

Table S4. Effect size comparing the relative abundance of the putative metabolic pathways in the roots versus that in the stem. A positive effect size indicates that the relative abundance was higher in the stem than in the roots while a negative value the opposite.

| ——————————————————————————————————————————— | | | | | |
| --- | --- | --- | --- | --- | --- |
|  |  | | | Relative abundance | |
|  | Effect size | | | —————— | |
|  | ——————— | | | Roots | Stem |
| Pathway | Con | Gly | Par | —— (%) —— | |
| ——————————————————————————————————————————— | | | | | |
| 5-aminoimidazole ribonucleotide biosynthesis I | 6.5 | 2.9 | 3.5 | 0.579 | 0.894 |
| 5-aminoimidazole ribonucleotide biosynthesis II | 6.4 | 2.8 | 3.3 | 0.569 | 0.917 |
| 6-hydroxymethyl-dihydropterin diphosphate biosynthesis I | 6.4 | 2.8 | 3.4 | 0.547 | 0.916 |
| 6-hydroxymethyl-dihydropterin diphosphate biosynthesis III (Chlamydia) | 6.3 | 2.7 | 3.4 | 0.525 | 0.803 |
| acetylene degradation | 5.0 | 2.1 | 3.1 | 0.619 | 1.129 |
| adenosine deoxyribonucleotides de novo biosynthesis II | 4.1 | 2.1 | 3.4 | 0.661 | 0.973 |
| adenosine ribonucleotides de novo biosynthesis | 5.3 | 2.1 | 3.4 | 0.678 | 0.984 |
| aerobic respiration I (cytochrome c) | 6.6 | 2.8 | 3.4 | 1.366 | 2.235 |
| Bifidobacterium shunt | 5.4 | 2.4 | 3.2 | 0.457 | 0.866 |
| Calvin-Benson-Bassham cycle | 6.2 | 2.2 | 3.5 | 0.672 | 1.021 |
| CDP-diacylglycerol biosynthesis I | 6.3 | 2.0 | 3.3 | 0.742 | 0.982 |
| CDP-diacylglycerol biosynthesis II | 6.1 | 2.1 | 3.3 | 0.742 | 0.982 |
| chorismate biosynthesis from 3-dehydroquinate | 5.4 | 2.1 | 3.4 | 0.624 | 0.860 |
| chorismate biosynthesis I | 5.5 | 2.2 | 3.3 | 0.651 | 0.923 |
| cis-vaccenate biosynthesis | 6.0 | 2.1 | 3.4 | 0.863 | 1.073 |
| coenzyme A biosynthesis I | 5.6 | 2.6 | 3.3 | 0.490 | 0.823 |
| colanic acid building blocks biosynthesis | 5.7 | 2.6 | 3.2 | 0.493 | 0.916 |
| dTDP-L-rhamnose biosynthesis I | 6.3 | 2.9 | 3.3 | 0.487 | 0.803 |
| dTDP-N-acetylthomosamine biosynthesis | 5.3 | 2.7 | 3.3 | 0.335 | 0.799 |
| fatty acid elongation -- saturated | 5.9 | 2.5 | 3.4 | 0.740 | 0.962 |
| flavin biosynthesis I (bacteria and plants) | 6.3 | 2.8 | 3.4 | 0.505 | 0.820 |
| GDP-mannose biosynthesis | 6.4 | 2.6 | 3.2 | 0.629 | 0.977 |
| gluconeogenesis I | 5.6 | 2.5 | 3.4 | 0.613 | 1.094 |
| glucose and glucose-1-phosphate degradation | 5.8 | 2.5 | 3.4 | 0.411 | 0.821 |
| glycogen biosynthesis I (from ADP-D-Glucose) | 5.4 | 2.2 | 3.3 | 0.549 | 0.946 |
| glycogen degradation I (bacterial) | 5.7 | 2.6 | 3.4 | 0.539 | 1.098 |
| glycolysis I (from glucose 6-phosphate) | 5.7 | 2.3 | 3.4 | 0.632 | 1.117 |
| glycolysis II (from fructose 6-phosphate) | 5.6 | 2.4 | 3.3 | 0.592 | 1.117 |
| glycolysis III (from glucose) | 4.9 | 2.1 | 3.5 | 0.601 | 0.967 |
| gondoate biosynthesis (anaerobic) | 6.2 | 2.0 | 3.5 | 0.839 | 0.988 |
| guanosine deoxyribonucleotides de novo biosynthesis II | 4.5 | 2.2 | 3.3 | 0.661 | 0.973 |
| guanosine ribonucleotides de novo biosynthesis | 5.0 | 2.0 | 3.2 | 0.629 | 0.870 |
| heme biosynthesis I (aerobic) | 6.1 | 2.8 | 3.4 | 0.452 | 0.801 |
| heme biosynthesis II (anaerobic) | 6.2 | 2.8 | 3.4 | 0.529 | 0.921 |
| inosine-5'-phosphate biosynthesis I | 6.7 | 2.7 | 3.5 | 0.486 | 0.809 |
| inosine-5'-phosphate biosynthesis III | 6.6 | 2.8 | 3.4 | 0.482 | 0.809 |
| L-arginine biosynthesis I (via L-ornithine) | 6.6 | 2.9 | 3.4 | 0.478 | 0.820 |
| L-arginine biosynthesis II (acetyl cycle) | 7.0 | 2.9 | 3.5 | 0.530 | 0.845 |
| L-arginine biosynthesis IV (archaebacteria) | 6.6 | 3.0 | 3.4 | 0.476 | 0.817 |
| L-glutamate and L-glutamine biosynthesis | 5.3 | 2.6 | 3.3 | 0.424 | 0.880 |
| L-histidine biosynthesis | 6.5 | 2.8 | 3.4 | 0.485 | 0.803 |
| L-isoleucine biosynthesis I (from threonine) | 6.6 | 2.8 | 3.5 | 0.721 | 0.968 |
| L-isoleucine biosynthesis II | 6.8 | 3.0 | 3.5 | 0.769 | 1.052 |
| L-isoleucine biosynthesis III | 6.5 | 2.7 | 3.6 | 0.634 | 0.929 |
| L-lysine biosynthesis III | 6.6 | 2.7 | 3.4 | 0.571 | 0.818 |
| L-lysine biosynthesis VI | 6.8 | 2.9 | 3.4 | 0.554 | 0.816 |
| L-ornithine biosynthesis | 6.8 | 2.9 | 3.4 | 0.502 | 0.803 |
| ——————————————————————————————————————————— | | | | | |

Table S4. Continued.

| ——————————————————————————————————————————— | | | | | |
| --- | --- | --- | --- | --- | --- |
| L-tryptophan biosynthesis | 6.3 | 2.7 | 3.5 | 0.528 | 0.893 |
| L-valine biosynthesis | 6.6 | 2.8 | 3.3 | 0.721 | 0.968 |
| methylerythritol phosphate pathway I | 5.6 | 2.1 | 3.4 | 0.564 | 0.856 |
| methylerythritol phosphate pathway II | 6.2 | 2.1 | 3.4 | 0.564 | 0.856 |
| mixed acid fermentation | 5.6 | 2.7 | 3.4 | 0.556 | 1.018 |
| NAD biosynthesis I (from aspartate) | 5.6 | 2.7 | 3.4 | 0.447 | 0.816 |
| nitrate reduction VI (assimilatory) | 4.9 | 2.7 | 3.2 | 0.323 | 0.954 |
| O-antigen building blocks biosynthesis (*E. coli*) | 5.9 | 2.5 | 3.3 | 0.498 | 0.823 |
| pantothenate and coenzyme A biosynthesis I | 6.4 | 2.7 | 3.4 | 0.478 | 0.814 |
| pentose phosphate pathway | 5.9 | 2.4 | 3.3 | 0.608 | 0.916 |
| pentose phosphate pathway (non-oxidative branch) | 5.4 | 2.2 | 3.4 | 0.795 | 0.983 |
| peptidoglycan biosynthesis I (meso-diaminopimelate containing) | 6.3 | 2.7 | 3.4 | 0.499 | 0.845 |
| peptidoglycan biosynthesis III (mycobacteria) | 6.0 | 2.6 | 3.4 | 0.496 | 0.845 |
| peptidoglycan maturation (meso-diaminopimelate containing) | 5.9 | 2.9 | 3.3 | 0.580 | 1.331 |
| phosphatidylglycerol biosynthesis I (plastidic) | 5.8 | 2.0 | 3.5 | 0.662 | 0.936 |
| phosphatidylglycerol biosynthesis II (non-plastidic) | 5.6 | 2.1 | 3.3 | 0.662 | 0.936 |
| phosphopantothenate biosynthesis I | 6.3 | 2.8 | 3.3 | 0.495 | 0.811 |
| preQ0 biosynthesis | 6.2 | 2.8 | 3.4 | 0.483 | 0.915 |
| pyrimidine deoxyribonucleotide phosphorylation | 5.6 | 2.7 | 3.3 | 0.482 | 0.823 |
| pyruvate fermentation to isobutanol (engineered) | 6.8 | 2.8 | 3.3 | 1.126 | 1.150 |
| queuosine biosynthesis | 5.5 | 2.1 | 3.4 | 0.546 | 0.856 |
| starch degradation V | 4.6 | 2.2 | 3.5 | 0.570 | 0.987 |
| sucrose degradation III (sucrose invertase) | 5.4 | 2.6 | 3.3 | 0.388 | 0.823 |
| sucrose degradation IV (sucrose phosphorylase) | 5.5 | 2.6 | 3.4 | 0.392 | 0.823 |
| sulfate reduction I (assimilatory) | 6.7 | 2.8 | 3.3 | 0.529 | 0.803 |
| superpathway of heme biosynthesis from glutamate | 6.4 | 2.8 | 3.4 | 0.457 | 0.805 |
| superpathway of 5-aminoimidazole ribonucleotide biosynthesis | 6.1 | 2.7 | 3.5 | 0.569 | 0.917 |
| superpathway of adenosine nucleotides de novo biosynthesis I | 5.4 | 2.1 | 3.3 | 0.698 | 1.013 |
| superpathway of adenosine nucleotides de novo biosynthesis II | 5.2 | 2.0 | 3.4 | 0.668 | 0.974 |
| superpathway of aromatic amino acid biosynthesis | 5.7 | 2.1 | 3.3 | 0.677 | 0.973 |
| superpathway of branched amino acid biosynthesis | 6.6 | 2.8 | 3.5 | 0.681 | 0.987 |
| superpathway of GDP-mannose-derived O-antigen building blocks biosynthesis | 6.6 | 2.8 | 3.3 | 0.509 | 0.909 |
| superpathway of geranylgeranyl diphosphate biosynthesis II (via MEP) | 5.6 | 2.5 | 3.3 | 0.466 | 0.831 |
| superpathway of guanosine nucleotides de novo biosynthesis I | 5.6 | 2.6 | 3.3 | 0.579 | 0.914 |
| superpathway of guanosine nucleotides de novo biosynthesis II | 5.4 | 2.5 | 3.2 | 0.566 | 0.895 |
| superpathway of L-alanine biosynthesis | 6.5 | 2.8 | 3.3 | 0.532 | 0.920 |
| superpathway of L-aspartate and L-asparagine biosynthesis | 4.5 | 2.0 | 3.2 | 0.538 | 0.931 |
| superpathway of L-isoleucine biosynthesis I | 6.6 | 2.9 | 3.4 | 0.583 | 0.869 |
| superpathway of L-serine and glycine biosynthesis I | 6.4 | 2.8 | 3.3 | 0.656 | 0.940 |
| superpathway of L-threonine biosynthesis | 6.4 | 2.7 | 3.4 | 0.529 | 0.814 |
| superpathway of phospholipid biosynthesis I (bacteria) | 6.5 | 2.0 | 3.2 | 0.694 | 0.954 |
| superpathway of purine nucleotides de novo biosynthesis I | 6.1 | 2.6 | 3.4 | 0.562 | 0.905 |
| superpathway of pyrimidine nucleobases salvage | 5.9 | 2.1 | 3.2 | 0.730 | 1.141 |
| superpathway of pyrimidine ribonucleotides de novo biosynthesis | 6.3 | 2.8 | 3.3 | 0.577 | 0.972 |
| TCA cycle VI (obligate autotrophs) | 6.5 | 2.8 | 3.4 | 0.515 | 0.851 |
| tetrapyrrole biosynthesis I (from glutamate) | 6.9 | 2.8 | 3.4 | 0.487 | 0.808 |
| tetrapyrrole biosynthesis II (from glycine) | 6.3 | 2.9 | 3.5 | 0.480 | 0.802 |
| thiamin salvage II | 6.7 | 2.8 | 3.4 | 0.476 | 0.803 |
| tRNA charging | 5.1 | 2.1 | 3.4 | 0.575 | 0.899 |
| tRNA processing | 5.6 | 2.6 | 3.4 | 0.454 | 0.822 |
| UDP-N-acetyl-D-glucosamine biosynthesis I | 5.2 | 2.1 | 3.3 | 0.585 | 0.857 |
| UDP-N-acetylmuramoyl-pentapeptide biosynthesis I (meso-diaminopimelate containing) | 6.8 | 2.8 | 3.5 | 0.505 | 0.856 |
| UDP-N-acetylmuramoyl-pentapeptide biosynthesis II (lysine-containing) | 6.4 | 2.8 | 3.6 | 0.495 | 0.838 |
| UMP biosynthesis | 6.7 | 2.8 | 3.4 | 0.578 | 0.963 |
| ——————————————————————————————————————————— | | | | | |
